# Hepatic alveolar echinococcosis in a child

**DOI:** 10.1590/0037-8682-0487-2020

**Published:** 2021-03-08

**Authors:** Bahar Yilmaz-Cankaya, Berhan Pirimoglu

**Affiliations:** 1 Ataturk University, Medical Faculty, Department of Radiology, Erzurum, Turkey.

**FIGURE 1:**
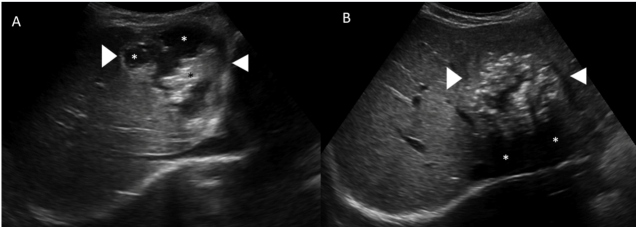
Transverse ultrasonography images **(A, B)** show a heterogeneous mass lesion (white arrowheads) that contained cystic components **(A, white asterisks**) and hyperechoic foci, representing calcifications **(A, black asterisk; B, white asterisk: shadows of the calcifications).**

**FIGURE 2:**
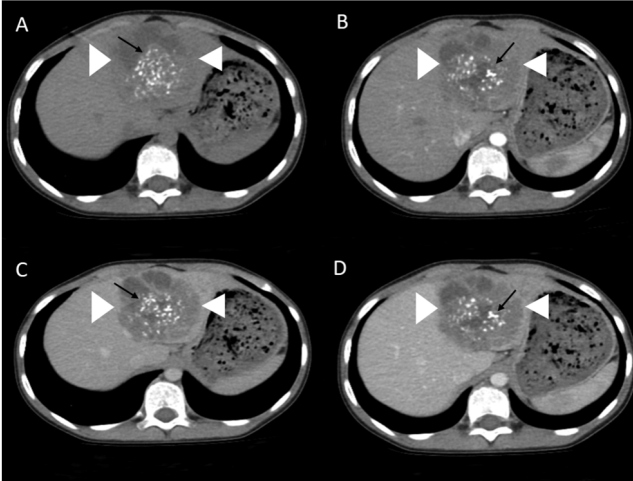
Axial non-contrast enhanced **(A)**, arterial phase **(B)**, portal phase **(C)** and venous phase **(D)** contrast enhanced multidetector computed tomography images show the hepatic alveolar echinococcosis lesion (white arrowheads) with irregular margins and heterogeneous cystic contents with scattered hyperattenuating foci, representing calcifications (black arrows)

A 10-year-old girl had a history of epigastric pain for three months. A laboratory investigation revealed mild leukocytosis with eosinophilia. Abdominal gray-scale ultrasonography showed a heterogeneous mass lesion in the left lobe of the liver. The mass was generally hypoechoic; however, it contained cystic components and hyperechoic foci, representing calcifications (Figure 1). Multidetector computed tomography (MDCT) demonstrated an infiltrating hepatic mass with irregular margins and heterogeneous cystic contents with scattered hyperattenuating foci, representing calcifications (Figure 2). The radiological imaging features and results of the immunoserological analyses (with a high sensitivity for Em2, a species-specific native antigen isolated from the metacestode of *Echinococcus multilocularis*), determined that she had hepatic alveolar echinococcosis (AE). The patient underwent surgical resection, and hepatic AE was confirmed by a histopathological examination. 

AE is caused by infection with the larvae of *E. multilocularis*. The correct identification of hepatic AE allows for appropriate treatment. Abdominal ultrasonography is the first-line imaging technique for the evaluation of patients in whom the presence of AE is suspected. MDCT and magnetic resonance imaging techniques could be performed for a preoperative evaluation. Liver lesions are seen as heterogeneous cystic-necrotic masses with calcifications on ultrasonography and MDCT. Hepatic AE lesions usually have irregular margins, scattered calcifications, central necrosis, and no significant enhancement in MDCT images^1^ ^,^ ^2^. In conclusion, hepatic AE is rare in children. However, this infection should be accounted for, especially in patients from endemic areas. 
